# Validity and reliability of the Polish version of the Pregnancy Mobility Index (PMI-PL)

**DOI:** 10.3389/fpubh.2024.1443616

**Published:** 2024-10-09

**Authors:** Katarzyna Antosiak-Cyrak, Joanna Ratajczak, Magdalena Lewandowska, Krystian Wochna, Katarzyna Sobczak, Katarzyna Domaszewska, Patrycja Rąglewska, Piotr Urbański, Urszula Czerniak, Anna Demuth

**Affiliations:** ^1^Department of Swimming and Water Lifesaving, Faculty of Sport Sciences, Poznan University of Physical Education, Poznań, Poland; ^2^Department of Human Biological Development, Faculty of Sport Sciences, Poznan University of Physical Education, Poznań, Poland; ^3^Calculation Centre, Poznan University of Physical Education, Poznań, Poland; ^4^Department of Physiology and Biochemistry, Faculty of Health Sciences, Poznan University of Physical Education, Poznań, Poland; ^5^Department of Physical Therapy and Sports Recovery, Faculty of Health Sciences, Poznan University of Physical Education, Poznań, Poland; ^6^Department of Adapted Physical Activity, Faculty of Sport Sciences, Poznan University of Physical Education, Poznań, Poland

**Keywords:** pregnant women, mobility limitation, women’s health services, lumbosacral region, PMI

## Abstract

**Introduction:**

Mobility, defined as active, controlled, multi-joint flexibility used in movement, is limited in pregnant women due to problems with low back pain (LBP) and pelvic girdle pain (PGP). The Pregnancy Mobility Index (PMI) is a tool for assessing mobility in relation to LBP/PGP. The lack of a Polish version of the PMI test prompted a transcultural adaptation to the Polish conditions. The aim of the study was to evaluate the measurement properties of the Polish adaptation of the Pregnancy Mobility Index.

**Methods:**

The study involved 121 pregnant women aged 18–44. The translation process was in accordance with the transcultural adaptation design. Reliability was assessed by intraclass correlation coefficient (ICC). Construct validity between the Polish version of the PMI (PMI-PL) and the Physical Activity Pregnancy Questionnaire (PPAQ-PL) was assessed by Spearman’s rank correlation coefficient.

**Results:**

The transcultural adaptation of the PMI test into Polish was satisfactory, with high internal consistency (Cronbach’s alpha = 0.97–0.98, ICC = 0.989). Statistically significant inverse proportional correlations were found for total PA, total PA (light and above), light PA, moderate PA, and vigorous PA in the construct validity analysis between PMI-PL and PPAQ-PL.

**Discussion:**

The Polish version of the PMI is a reliable instrument. The introduction of a questionnaire with a classification system will make it easier for health professionals to monitor the health status of pregnant women and encourage them to engage in physical activity appropriate for their current level of mobility.

## 1 Introduction

Physical activity (PA) in pregnant women is an issue that is well documented in the scientific literature. Despite this, pregnant women do not meet the guidelines for appropriate levels of physical activity. Previous studies by Antosiak-Cyrak and Demuth (1) and Demuth et al. (2) have shown that pregnant women in Poland prefer low to moderate intensity physical activity. Failure to achieve the recommended level of weekly physical activity can pose a serious threat to maternal well-being (3), which consequently becomes a public health problem and prompts interventions to monitor and educate women on this issue.

Evidence presented in the “2018 Physical Activity Guidelines Advisory Committee Scientific Report” (4) confirms the positive impact of regular physical activity before and during pregnancy on reducing the risk of a range of diseases and conditions, including gestational diabetes (5, 6), excessive weight gain (7, 8) and postpartum depression (9).

The 2020 World Health Organisation (WHO) recommendations (10) for the first time include new specific recommendations for pregnant and postpartum women. These recommendations include moderate-intensity aerobic activity of 150 min per week, which coincides with the 2018 American College of Obstetricians and Gynecologists (ACOG) guidelines for Americans and is equivalent to 500 MET min week^−1^ (11, 12). The consensus on recommendations for intensity, duration and monitoring of physical activity should make it easier for both the community and health professionals to promote physical activity consistently and substantively in each trimester of pregnancy (13, 14).

So why do women with a normal pregnancy not engage in physical activity as recommended by WHO, or even reduce the activity they undertook before pregnancy? McKeough et al. (15) identifies the physiological effects of pregnancy, lack of knowledge about safe activity during pregnancy and the beliefs of friends and family as the main barriers to physical activity, while Perera and Tinius (16) also identifies lack of time and lack of social support. The analysis of research on the socio-demographic determinants of physical activity in pregnant women by Sun et al. (17) also highlights the lack of organisational and political support for the promotion of regular physical activity during pregnancy.

Physiological symptoms characteristic of pregnancy, such as fatigue, morning sickness, disturbed sleep and lumbopelvic pain, are the main factors limiting physical activity (18–20). Pain between the iliac crests and the gluteal folds at the level of the sacroiliac joints, known as low back pain (LBP) and pelvic girdle pain (PGP), also contribute to limitations in household and work activities and mobility.

Health professionals, including gynaecologists, midwives and physiotherapists, should regularly monitor the physical activity levels of pregnant women, which would prevent their withdrawal from work (by reducing the need for sick leave) and social activity. Currently available tests to assess the level of physical activity in pregnant women are time-consuming. There is a need for an easy-to-use and straightforward tool to screen women’s mobility, such as the Pregnancy Mobility Index (PMI) by van de Pol et al. (21). The PMI is a tool used to assess mobility in relation to back and pelvic pain, but also to monitor training progress (22) and to assess the effectiveness of rehabilitation for pregnant women in relieving lumbar pain (23).

PMI has a good construct validity and also shows a strong association with lumbar pain, which occurs as the pregnancy progresses and as the quality of life, both emotional and physical, deteriorates (21, 24–26).

Regular administration of the PMI test during antenatal visits can help health professionals identify critical moments of deteriorating mobility and encourage engagement in physical activity, taking into account existing mobility limitations.

In Poland, there has been a lack of easy-to-use, reliable, and accurate tools for assessing the mobility of pregnant women, which has resulted in an inability to draw accurate conclusions and make cross-population comparisons. Given the need to develop research on the mobility of pregnant women, it was essential to either develop a new questionnaire or adapt an existing one to Polish conditions that would meet the methodological requirements. This was essential to ensure that Polish researchers had access to validated research tools. The aim of the study was to evaluate the measurement properties of the Polish adaptation of the Pregnancy Mobility Index (PMI-PL). It was hypothesized that the Polish version of the PMI (PMI-PL) would have similar reliability and relevance values to its original versions and would be an accurate questionnaire for assessing the mobility of pregnant women in the Polish population.

## 2 Methods

### 2.1 Translation and cultural adaptation

A cross-cultural adaptation design recommended by Beaton et al. (27) was used in this translation process. Permission was obtained from the creator of the test, van de Pol et al. (21), to conduct a cultural adaptation into the Polish language. The adaptation included forward and backward translation, the use of difficulty and quality scores, pilot testing and cross-cultural comparison of translations.

#### 2.1.1 Procedure

Stage I: Forward translation (English to Polish).

In the first stage, two researchers carried out the forward translation. The translators developed independent versions of the questionnaire, which were then compared and the results synthesised by the research team.

Stage II: Expert panel back-translation (Polish to English).

Without seeing the original version, two other researchers translated the final forward translated version into English. The back-translated version of the test was compared with the original. The research team then reviewed the initial translated version, further rephrasing and reformulating some elements to minimise any discrepancies from the original version and to adapt the translated version appropriately to the Polish culture.

Stage III: Cognitive interview (pre-testing).

A cognitive interview (pre-testing) was carried out with 32 patients to ensure the comprehensibility of PMI-PL. Final adjustments were made, which seemed to be important due to the cognitive aspects of the cultural issue in Poland. Following this, 121 pregnant women were asked to complete the finalized questionnaire (PMI-PL).

Stage IV: Final version.

**Figure 1:**
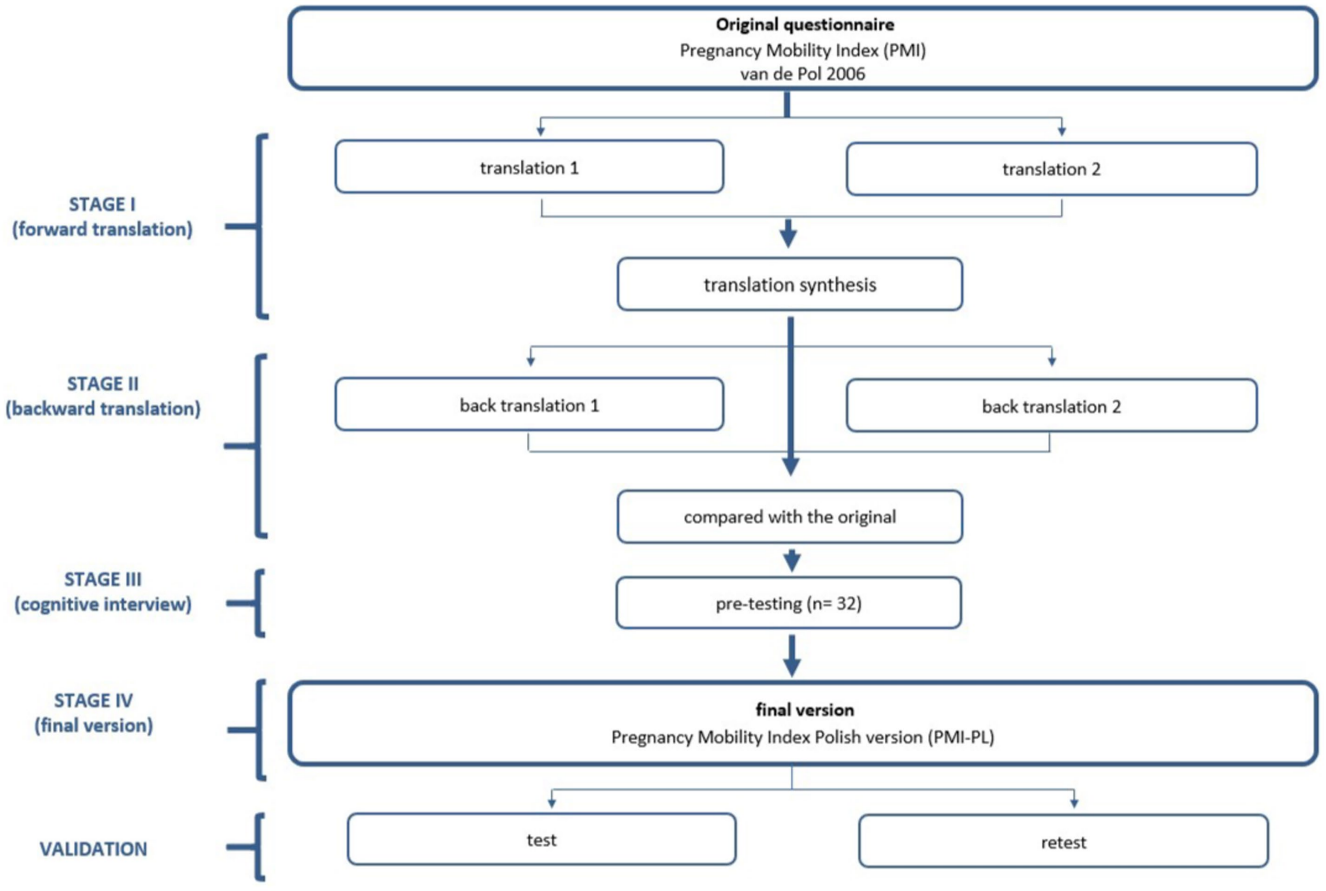
The flowchart of the study design.

The final version of the test is the result of all the iterations described above (Figure 1).

### 2.2 Participants

A total of 121 pregnant women participated in the research studies. Pregnancy stage, socio-demographic data such as education and place of residence, were recorded. The bioethics committee did not identify any characteristics of medical experimentation in the studies. The study was conducted in the Greater Poland Voivodeship (Poland) among patients at gynaecological and obstetric outpatient clinics during routine check-ups from September 2021 to June 2022. The inclusion criteria were as follows: a singleton pregnancy, good maternal and/or fetal health, age over 18 years, and no language barriers.

### 2.3 Questionnaires

#### 2.3.1 Pregnancy Mobility Index (PMI)

The PMI consists of 24 questions divided into three subscales: the first subscale includes questions about daily mobility at home (questions 1–7); the second subscale includes typical household activities (questions 8–16); the third subscale includes mobility outdoors (questions 17–24). Each question refers to pain in the pelvic floor muscles, which affects the mobility of pregnant women. Women answer on a 4-point scale: “no problem performing this task”; “some effort performing this task”; “much effort performing this task”; and “performing this task is impossible or only possible with the aid of others.” Each response is scored from 0 to 3, with higher scores indicating more severe impairment. The score is calculated by averaging the scale positions (0–100). The Pregnancy Mobility Test in its original version (21) shows internal consistency at a Cronbach’s alpha level of 0.80 or higher and is suitable for detecting changes in mobility during pregnancy.

#### 2.3.2 Pregnancy Physical Activity Questionnaire—PPAQ-PL

The Polish version of the PPAQ (28) was used to assess weekly energy expenditure (MET hours/week^−1^). Participants self-assessed their level of physical activity by completing a questionnaire consisting of 33 items categorised into the following types of activity: household/caregiving (15 items), occupational (5 items), sport/exercise (7–9 items), transportation (3 items), and inactivity (3 items). The declared duration of each tasks was assigned a fixed number of minutes (0, 0.12, 0.50, 1.0, 2.0, and 3.0) and then multiplied by the number of days per week that the task was performed. The obtained values were then multiplied by the intensity (MET) according to the guidelines in “Compendium of Physical Activities: an update of activity codes and MET intensities” (29). The following activity intensity ranges were used: sedentary <1.5 METs; light 1.5 - < 3.0 METs; moderate ≥3.0 - ≤ 6.0 METs; and vigorous >6.0 METs. In addition to the above, according to the calculation instructions of the PPQA questionnaire (28), the study distinguished categories such as “total PA” which is the sum of all activities, and “total PA (light and above),” which is the sum of low and higher intensity activities.

### 2.4 Statistical analysis

All the analyses were conducted using the TIBCO Software Inc. (2017) Statistica (data analysis software system; version 13; available at http://statistica.io) and the jamovi project (2021), jamovi [Version 2.2; (Computer Software); available at https://www.jamovi.org].

The significance cutoff value in this study was assumed at *p* < 0.05.

Quantitative variables were characterised using arithmetic mean ( $\bar{x})$ and standard deviation (SD), while qualitative variables were presented using counts (*n*) and percentages (%). The normality of distribution for quantitative variables was assessed using the Shapiro–Wilk test. The homogeneity of variance was assessed using the Levene’s test.

### 2.5 Reliability

The intraclass correlation coefficient (ICC) is a widely used reliability index in test-retest analyses. To assess the consistency of repeated measurements, the ICC (2, *k*) was used accordinf to the methodology proposed by Koo and Li (30). The ICC values were interpreted as follows: <0.50 was considered as poor reliability, ≥0.50 and <0.75 as moderate reliability, ≥0.75 and <0.90 as good reliability, and >0.90 as excellent reliability (31). The internal consistency of all PMI scales was assessed using Cronbach’s alpha. A Cronbach’s alpha between 0.70 and 0.95 was considered satisfactory (32).

#### 2.5.1 Construct validity

Construct validity was assessed using the Spearman’s rank correlation analysis to determine the association between the Italian version of the 5-degree intensity scale and the 4-degree life category scale of the PPAQ-PL and the three subscales of the PMI. We hypothesised that there would be a high degree of confidence between these two questionnaires. Coefficients <0.30, 0.30 to 0.60, and >0.60 were considered to indicate low, moderate, and high correlations, respectively (33).

#### 2.5.2 Cross-cultural analysis

The Kruskal–Wallis test (*H*) and the *U* Mann–Whitney test with continuity correction (*Z*) were used to identify factors influencing modifications in the test results. The use of non-parametric statistical tests was due to the abnormal distribution assumptions of the studied variables. Three PMI subscales and the total score were included in the analysis, adjusted for age, trimester, place of residence, and difference in body mass (current minus pre-pregnancy) of the surveyed women.

## 3 Result

### 3.1 Translation and cultural adaptation

#### 3.1.1 Polish version of PMI—PMI-PL

The Polish translation (Appendix A) required a minor change to question 8. There were suggestions from respondents to change the term “vacuum cleaning” to a more precise formulation “cleaning on the floor with a vacuum cleaner.” Both the original and the Polish version assess mobility in three categories of activities: daily mobility (items 1–7); household activities (items 8–16); mobility outdoors (items 17–24) and total score (items 1–24).

##### 3.1.1.1 Scoring

**Figure 2:**
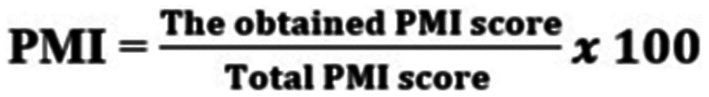
The PMI formula. The obtained PMI score = score achieved by a single individual; Total PMI score = sum of all points obtained in the tool equaling 72 (24 questions × 3 points).

Each question is scored on a scale of 0–3, where 0—no problems performing this task; 1—some effort performing this task; 2—much effort performing this task; 3—performing this task is impossible or only possible with the aid of others. The maximum score is 72 and failure to answer any question invalidates further analysis of the results. The final score represents a dimensional scale from 0 to 72 points. According to the author’s recommendation (21), the test results obtained should be expressed on a scale from 0 to 100 according to the formula shown in Figure 2 (the same formula in Polish is shown in Appendix A).

##### 3.1.1.2 PMI test classification

The original version of the test did not include a mobility classification. For epidemiological purposes, the development of such a classification seems justified. It will enable nurses, doctors and physiotherapists to assess the mobility of pregnant women and to recommend a safe type and intensity of exercise. It is therefore necessary to establish a problem category that is directly proportional to the number of points obtained.

There is a strong correlation between spinal pain symptoms as measured by the Oswestry Disability Index (ODI) and the PMI test (21), so a method similar to the ODI was used for both score calculation and group assignment (34) (Table 1).

**Table 1:** Classification of mobility in pregnant women—groups.

PMI groups	Point ranges	Category name
0	0	No mobility limitations whatsover
1	1–20	Very good mobility
2	21–40	Good mobility
3	41–60	Moderate mobility
4	61–80	Low mobility
5	81–100	Total lack of mobility

##### 3.1.1.3 Participants

The study included 121 pregnant women aged 18 to 44 years ( $\bar{x}$ = 28.9 ± 4.7) (Table 2). The largest group consisted of women aged 26–30 years (56.2%). The vast majority of women surveyed had higher education (63.6%) and lived in urban areas (66.1%). Almost 40% of the women were in their first trimester of pregnancy, one in four were in their second trimester and 35.5% were in their third trimester. More than half of the participants were first-time mothers.

**Table 2:** Group characteristics.

Variables	Total *n* = 121
Age % (*n*)	
<26 y.o.	21.5 (26)
26–30 y.o.	56.2 (68)
>30 y.o.	22.3 (27)
Trimester	
1st	39.7 (48)
2nd	24.8 (30)
3rd	35.5 (43)
Number of children	
0	54.6 (66)
1	25.6 (31)
≥2	19.8 (24)
Education	
Primary	12.4 (15)
Secondary	24.0 (29)
Higher	63.6 (77)
Place of residence	
Rural	33.9 (41)
Urban	66.1 (80)

The overall level of mobility of the women surveyed, as measured by the PMI-PL, averaged 19.20 ± 22.33 points (min = 0; max = 80.56) (Table 3). The lowest scores were recorded for questions in the domain of mobility outdoors ( $\bar{x}$ = 17.0 ± 21.2), while the highest scores were recorded in the domain of household activities ( $\bar{x}$ = 20.7 ± 24.2).

**Table 3:** Characterisation of mobility and activity of pregnant women using PMI-PL and PPAQ-PL.

Variables	$\bar{x}$ ± SD	Min–max
PMI-PL (points)		
Daily mobility	19.9 ± 24.0	0–90.4
Household activities	20.7 ± 24.2	0–85.2
Mobility outdoors	17.0 ± 21.2	0–79.2
Total score	19.2 ± 22.3	0–80.6
PPAQ-PL (METs)		
Total PA	170.0 ± 98.5	40.2–686.0
Total PA (light-intensity and above)	157.0 ± 97.6	28.7–681.5
PPAQ-PL by intensity (METs)		
Sedentary PA	13.0 ± 11.9	0.8–58.8
Light PA	103.9 ± 49.9	11.4–239.7
Moderate PA	51.8 ± 63.8	1.6–462.5
Vigorous PA	1.4 ± 3.9	0–27.9
PPAQ-PL by type (METs)		
Household activities	79.4 ± 50.8	12.1–339.7
Occupational	43.0 ± 70.6	0–592.2
Sport/exercise	7.0 ± 9.3	0–53.9
Transportation	18.0 ± 13.9	0–85.8
Inactivity	22.6 ± 15.0	0.8–68.6

The mean total PA level of the women surveyed, as measured by the PPAQ-PL questionnaire, was 170.0 ± 98.5 METs (min = 40.2; max = 686.0), the mean total PA (average and above) was 157.0 ± 97.6 METs (min = 28.7; max = 681.5). Regarding the division of PA by intensity, the highest energy expenditure was recorded in the light PA category ( $\bar{x}$ = 103.9 ± 49.9 METs), which accounted for 61% of the total PA. Conversely, the lowest energy expenditure was observed for high intensity PA ( $\bar{x}$ = 1.4 ± 3.9), which accounted for 0.8% of the total PA. Regarding the classification of activities by type, the highest energy expenditure was recorded for household activities ( $\bar{x}$ = 79.4 ± 50.8), which accounted for almost half of the total PA (46.7%), while the lowest energy expenditure was recorded for sport and exercise ( $\bar{x}$ = 7.0 ± 9.3), which accounted for only 4.1% of the total PA.

The pregnant women surveyed differed in terms of their mobility (Table 4). No mobility limitations and very good mobility characterised 61.2% of the participants. Low mobility was reported by a small percentage of respondents (5%), while complete lack of mobility was reported by only one participant (0.8%).

**Table 4:** Classification of mobility in pregnant women.

PMI groups	Total PMI score % (*n*)
0	30.6 (37)
1	30.6 (37)
2	19.8 (24)
3	13.2 (16)
4	5.0 (6)
5	0.8 (1)

##### 3.1.1.4 Test reliability

The reliability of the test, as measured by Cronbach’s alpha, showed excellent internal consistency. Cronbach’s alpha for both test items ranged from 0.88 to 0.98. The internal consistency (ICC) ranged from 0.997 to 0.999 (Table 5).

**Table 5:** Transcultural adaptation of PMI-PL.

Cronbach’s alpha		Interclass correlation coefficient		
Term 1	Term 2	ICC (2.k)	95% CI lower	95% CI upper
0.96	0.95	0.998	0.997	0.999
0.96	0.95	0.997	0.996	0.998
0.92	0.88	0.999	0.998	0.999
0.98	0.97	0.989	0.984	0.992

##### 3.1.1.5 Construct validity

Spearman’s rank correlation showed statistically significant relationships between most categories of physical activity (PPAQ-PL) and type of mobility (PMI-PL). Inversely proportional relationships were observed for most of the results (Table 6).

**Table 6:** Construct validity—relationship between PPAQ-PL and PMI-PL.

	PMI-PL			
PPAQ-PL	Daily mobility	Household activities	Mobility outdoors	Total score
	Spearman’s rank correlation coefficient (*p*-value)			
PPAQ-PL				
Total PA	**−0.40 (<0.001)**	**−0.32 (<0.001)**	**−0.28 (0.002)**	**−0.35 (<0.001)**
Total PA (light-intensity and above)	**−0.42 (<0.001)**	**−0.34 (<0.001)**	**−0.30 (0.001)**	**−0.37 (<0.001)**
PPAQ-PL by intensity				
Sedentary PA	0.14 (0.128)	0.14 (0.135)	0.17 (0.063)	0.16 (0.083)
Light PA	**−0.32 (<0.001)**	**−0.27 (0.003)**	**−0.22 (0.015)**	**−0.28 (0.002)**
Moderate PA	**−0.40 (<0.001)**	**−0.29 (0.001)**	**−0.27 (0.003)**	**−0.32 (<0.001)**
Vigorous PA	**−0.25 (0.006)**	**−0.25 (0.005)**	**−0.27 (0.002)**	**−0.27 (0.003)**
PPAQ-PL by type				
Household activities	−0.14 (0.135)	−0.08 (0.408)	−0.01 (0.923)	−0.08 (0.390)
Occupational	**−0.53 (<0.001)**	**−0.47 (<0.001)**	**−0.47 (<0.001)**	**−0.49 (<0.001)**
Sport/exercise	−0.05 (0.595)	−0.07 (0.445)	−0.06 (0.496)	−0.08 (0.372)
Transportation	−0.15 (0.092)	−0.08 (0.371)	−0.11 (0.214)	−0.13 (0.158)
Inactivity	0.16 (0.078)	**0.18 (0.043**)	**0.20 (0.033)**	0.18 (0.051)

*p-value <0.05—a statistically significant value. The bold values used to highlight statistically significant results.*

##### 3.1.1.6 Cross-cultural analysis

Cross-cultural analysis was performed to determine if there were differences in PMI-PL scores within subgroups of the population. Statistical differences were found for trimester of pregnancy and place of residence (Table 7). Women in the first trimester of pregnancy had the best mobility scores in each of the subscales analysed. The greatest mobility limitations were observed in women in the third trimester of pregnancy and in urban dwellers. No significant associations were found between PMI-PL and age or PMI-PL and the number of children.

**Table 7:** Cross-cultural analysis.

PMI categories	Variables	$\bar{x}$ ± SD	*H*/*Z*	*p*-value
Age				
Daily mobility	18–25 y.o.	23.63 ± 23.73	2.25^a^	0.325
	26–30 y.o.	17.65 ± 23.79		
	>30 y.o.	21.87 ± 24.89		
Household activities	18–25 y.o.	26.21 ± 25.66	1.27^a^	0.529
	26–30 y.o.	19.34 ± 24.14		
	>30 y.o.	18.66 ± 23.07		
Mobility outdoors	18–25 y.o.	21.79 ± 24.28	1.89^a^	0.389
	26–30 y.o.	15.20 ± 20.15		
	>30 y.o.	16.82 ± 20.69		
Total score	18–25 y.o.	23.99 ± 23.75	2.10^a^	0.350
	26–30 y.o.	17.46 ± 22.00		
	>30 y.o.	18.98 ± 21.91		
Trimester				
Daily mobility	1st	1.98 ± 4.26	67.85^a^	**<0.001**
	2nd	19.37 ± 21.80		
	3rd	40.20 ± 22.44		
Household activities	1st	2.39 ± 5.27	66.64^a^	**<0.001**
	2nd	22.10 ± 21.91		
	3rd	40.05 ± 23.34		
Mobility outdoors	1st	3.21 ± 7.46	55.57^a^	**<0.001**
	2nd	17.22 ± 21.10		
	3rd	32.17 ± 21.47		
Total score	1st	2.55 ± 5.05	64.54^a^	**<0.001**
	2nd	12.76 ± 17.31		
	3rd	36.54 ± 23.31		
Number of children				
Daily mobility	0	24.24 ± 25.29	5.10^a^	0.078
	1	16.90 ± 22.70		
	≥2	11.71 ± 19.51		
Household activities	0	25.31 ± 25.92	5.64^a^	0.060
	1	18.16 ± 22.23		
	≥2	11.11 ± 18.73		
Mobility outdoors	0	20.14 ± 23.21	3.34^a^	0.188
	1	14.65 ± 17.54		
	≥2	11.28 ± 18.69		
Total score	0	23.27 ± 23.99	3.84^a^	0.147
	1	16.62 ± 19.69		
	≥2	11.34 ± 18.64		
Place of residence				
Daily mobility	Rural	13.24 ± 19.59	−2.07^b^	**0.038**
	Urban	23.27 ± 25.37		
Household activities	Rural	15.63 ± 23.72	−2.03^b^	**0.043**
	Urban	23.24 ± 24.20			PMI categories	Variables	*x* ± SD	*H*/*Z*	*p*-value
Mobility outdoors	Rural	12.50 ± 19.96	−1.98^b^	**0.048**
	Urban	19.27 ± 21.56		
Total score	Rural	13.89 ± 20.62	−1.90^b^	0.058
	Urban	21.93 ± 22.80		

a ANOVA Kruskal–Wallis (*H*).

b *U* Mann–Whitney test with continuity correction (*Z*); *p*-value <0.05—a statistically significant value. The bold values used to highlight statistically significant results.

## 4 Discussion

The transcultural adaptation of the PMI test into Polish proved to be satisfactory. The results showed a high internal consistency. Cronbach’s alpha for the test ranged from 0.97 to 0.98, with an intraclass correlation coefficient (ICC) of 0.989. For questions related to “Daily mobility” at home, Cronbach’s alpha ranged from 0.95 to 0.96, with an ICC of 0.998, for questions related to “Household activities” it ranged from 0.95 to 0.96, with an ICC of 0.997, while the last group of questions related to “Mobility outdoors” had Cronbach’s alpha values of 0.88 to 0.92, with an ICC of 0.999. These results are in line with the original test by van de Pol et al. (21), where the internal consistency of the total score (Cronbach’s alpha) was >0.8. In the Italian version the total score was 0.945 (24) and in the Brazilian Portuguese version it was >0.90 (25). The structure of the questions is simple and does not raise methodological issues arising from the translation and transcription of the text in the present adaptation. There was only one suggestion from respondents to change the term “vacuum cleaning” to a more precise formulation such as “cleaning on the floor with a vacuum cleaner.” A similar change was made in the Manzotti et al. (24).

It was decided to analyse the validity of the PMI-PL construction by means of a questionnaire measuring physical activity (PPAQ-PL) (28). Undoubtedly, mobility encompasses different categories of life and intensities of activities undertaken. The cultural adaptation of the original version by Chasan-Taber et al. (35), which has been carried out in many populations, including Polish (28, 36), makes it possible to compare the physical activity of pregnant women in the life areas and intensities defined by the test. In this study, statistically significant inverse proportional correlations were found for total PA, total PA (light and above), light PA, moderate PA, and vigorous PA in the construct validity analysis between PMI-PL and PPAQ-PL. The absence of pain symptoms in the lumbopelvic-hip complex, such as pain between the iliac crest and the gluteal folds at the level of the sacroiliac joints, allows pregnant women to move freely, which is reflected in the intensity of effort and the performance of work, with which significant relationships were also observed. No significant relationship was found between sedentary activity and the PMI-PL subscales analysed. No association was found between PMI-PL and areas of life such as housework or mobility, which can be explained by the need to carry out housework regardless of the discomfort caused by mobility.

The cross-cultural analysis of the Polish adaptation of the PMI showed a direct proportional relationship between mobility and the trimester of pregnancy, which is consistent with findings in the literature (37), as well as with the place of residence. In the study by Manzotti et al. (24), an association was found between BMI and PMI, as well as between the place of residence and PMI. Women with lower body mass living in rural areas were found to have greater pain symptoms during mobility in every aspect analysed (PMI). Living and working in a rural environment is associated with stress in a standing position, which according to Ceprnja et al. (26) is a risk factor for pelvic complaints. In our own research, women living in urban areas experienced more pain symptoms in the lumbopelvic-hip region, which was directly related to the average PMI score in the subscales analysed. Further research is needed to explain these associations.

Physical activity during pregnancy is essential for the proper development of the baby and for the mother’s health. Current research suggests that the level of physical activity among pregnant women is dangerously low. The mean total activity score obtained in this study (PPAQ-PL = 170.02 ± 98.51) is far from sufficient, but typical for the population of pregnant women in Poland, as confirmed by previous studies by Antosiak-Cyrak and Demuth (1), as well as by Krzepota and Sadowska (28) and Wojtyła et al. (38). This confirms the need to extend the recommendations of the Polish Gynaecological Society (39), possibly by conducting screening tests with PMI-PL. Increased lower back pain during pregnancy significantly limits mobility, which can significantly affect the level of physical activity in pregnant women. Knowledge of barriers to physical activity during pregnancy can assist perinatal care and influence adherence to physical activity guidelines during pregnancy by adapting a flexible, individualised plan of action to the physical changes that occur during pregnancy. This knowledge will help antenatal care providers to develop physical activity interventions for pregnant women that meet individual needs, optimise enabling factors and overcome barriers to behaviour change from intention to action.

Despite the widespread prevalence of pregnancy-related lumbopelvic pain, it is often under-reported by women (40), and few seek effective treatment (41). This has significant physical, psychosocial, and economic consequences. The practical implications of the results of this study are significant for the care of pregnant women and may help to improve the quality of this care in Poland. Regular monitoring of mobility using the Polish Pregnancy Mobility Index (PMI-PL) during prenatal visits can help identify when a woman’s mobility begins to decline. This allows healthcare professionals to implement appropriate interventions, including rehabilitation and physiotherapy, to address mobility problems and to assess the effectiveness of these treatments (42). The PMI-PL can also identify problematic aspects of physical activity and areas where pregnant women need support, so that exercise plans can be tailored to their changing needs throughout pregnancy (43). It can also be used in population studies to assess and compare mobility in different regions of Poland. To summarise, regular use of the PMI-PL could improve prenatal care by increasing the detection of mobility problems and the effectiveness of preventive and therapeutic measures, which are crucial for the well-being of pregnant women.

The study has some limitations that need to be taken into account. The Polish version of the PMI (PMI-PL) was adapted on a sample of pregnant women within a specified age range, excluding those under 18 years of age. Most of the women surveyed were pregnant for the first time, had a high level of education, and lived in large cities, which does not fully reflect the general demographic structure of Poland.

## 5 Conclusion

The transcultural adaptation of the PMI test into Polish proved to be satisfactory. The tool showed good reliability, with an overall Cronbach’s alpha value between 0.88–0.98. The ICC coefficient was between 0.997 and 0.999. The PMI-PL appears to be a reliable and relevant questionnaire for assessing the mobility of pregnant women, so that reliable conclusions can be drawn from subsequent studies using this tool. The authors hope that the implementation of the classification questionnaire will enable a significant number of midwives, gynaecologists and physiotherapists to monitor the health status of pregnant women and encourage them to undertake physical activity appropriate to their current mobility.

## Figures and Tables

**Figure A1:**
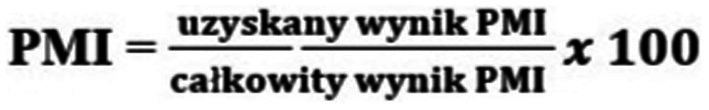
The PMI formula in Polish. Gdzie: Uzyskany wynik PMI = wynik uzyskany przez pojedynczą osobę, Całkowity wynik PMI = suma wszystkich punktów uzyskanych w narzędziu równa 72 (24 pytania × 3 punkty).

## Data Availability

The raw data supporting the conclusions of this article will be made available by the authors, without undue reservation.

## Appendix A

The tool presented in this appendix is the validated Polish version of the Pregnancy Mobility Index, originally developed by van de Pol. The tool is designed to assess mobility in relation to low back pain and pelvic girdle pain.

### KWESTIONARIUSZ OCENY MOBILNOŚCI KOBIET W CIĄŻY—PMI-PL

Czy doświadczasz ograniczeń lub uskarżasz się na dolegliwości w okolicy obręczy biodrowej lub kręgosłupa w trakcie wykonywania następujących czynności:

**Table:**

Ocena mobilności w trakcie wykonywania codziennych czynności domowych					
l.p	Pytania	0 Bez Problemu	1 Pewien Wysiłek	2 Duży Wysiłek	3 Niemożliwe/z inną osobą
Czynności codzienne:					
1	Wstawanie z twardego krzesła				
2	Wstawanie z miękkiego krzesła				
3	Wstawanie z łóżka				
4	Podnoszenie przedmiotów z podłogi				
5	Zakładanie butów				
6	Przewracanie się na bok w łóżku				
7	Wstawanie z podłogi				
Czynności podczas prac domowych:					
8	Odkurzanie za pomocą odkurzacza				
9	Pranie w pralce				
10	Wieszanie prania				
11	Prace na kolanach				
12	Prace w przysiadzie				
13	Prace na stojąco				
14	Podnoszenie 5 kg				
15	Podnoszenie 10 kg				
16	Wchodzenie po schodach				
Czynności poza domem:					
17	Podróż pociągiem				
18	Podróż samochodem				
19	Jazda rowerem				
20	Podróż autobusem				
21	Pokonanie 50 m pieszo				
22	Pokonanie 200 m pieszo				
23	Pokonanie 500 m pieszo				
24	Chodzenie po nierównym terenie				

Punktacja odpowiedzi na pytania w kwestionariuszu: “Czynność wykonywana bez problemów”—0 pkt; “Wykonanie czynności wymaga pewnego wysiłku”—1 pkt; “Wykonanie czynności wymaga dużego wysiłku”—2 pkt; “Wykonanie tej czynności jest niemożliwe lub możliwe tylko z pomocą innych osób”—3 pkt. Uzyskane wyniki testu powinny być wyrażone w skali od 0 do 100 zgodnie ze wzorem przedstawionym na Figure A1.
